# A Proper Food Standard in Its Relation to the Teeth

**Published:** 1899-09

**Authors:** George R. Gray

**Affiliations:** Worcester, Mass.


### A PROPER FOOD STANDARD IN ITS RELATION TO THE TEETH.[1]

BY GEORGE R. GRAY, D.D.S., D.M.D., WORCESTER, MASS.

One of the most important subjects from the stand-point of the dentist must of necessity be, What is the best article of nourishment that can be used to help give added strength to that part of our system in which we are most interested?

From the few investigations that have been made in the public schools of our country, the alarming fact is known to us that there is a most deplorable condition to be found in the mouths of the little ones. As we know that this is partly due to the neglect of these important organs, still the fact remains that it cannot be wholly due to this, for if we examine into the habits of the children, we are sure to find that the food they eat is in a great measure composed of that which by no stretch of the imagination can be called nourishing.

Our business is mainly that of a restorer and repairer of dental organs, but if we are to be entirely honest to ourselves and patients, our duty cannot end at that point. We have been handicapped in our efforts by the social problems surrounding us, and have not given as much time to dietary reform as we should have done. We must find a preventive, and this can only come through perfect physical development of the entire body, and it is along this line that we must concentrate our efforts.

We develop and subsist solely upon such substances as are taken into the body by way of the mouth as foods. With this fact clear, it is plain that such foods must contain, in certain proportions, the exact chemical elements of which our bodies are composed, or perfect development will not take place. There is no question but that, taken as a nation, we are a candy-eating and prepared-food-consuming people, and this to a great extent is the cause of our poorly constructed teeth. The question might be asked, why we of to-day, comfortably clothed, well fed, apparently should have fallen so from the high standard of our grandparents? The scientist would say that we are starved, and he would be correct, for with food plenty and of the best, we throw away all that which makes muscle, bone, sinew, teeth, hair, etc., and in the barrel of snow-white best family

[1] Read before the Harvard Odontological Society, March 30, 1899.

flour they retain only the starchy elements that supply the body with heat, the carbonates.

Natural foods make possible natural conditions and build the harmonious body.

Our forefathers, whose food consisted mainly of coarsely ground grains and unbolted flour, were men of wonderful strength of fibre, intellectual, virile, and aggressive. Their families were large, and their descendants rapidly peopled the land. With their store-houses running over with grain, they introduced the bolting-cloth and used for their own food fine white flour from the heart of the grain. All the rest, the bone- and sinew-producing part, which could not be so finely ground and which gave the dark color, was thrown to the animals. Nature followed her unvarying law and took her revenge. For those who would not eat the bone-building gluten she refused to supply bone-material, and as a natural result a full sound set of teeth became a thing almost unknown, and the frail, half-nourished apologies for teeth gave out in early youth.

That which most nearly furnishes in the correct proportions all the properties to nourish all the elements of the body is wheat that comes to the miller from the fields. Unfortunately, wheat as it is usually presented to us in the form of bread, biscuits, cake, etc., is, in the effort to produce the æsthetic effect of whiteness, robbed of its nitrates or phosphates, in which reside the bone- and muscle-producing elements, while there is left only the carbonates or heat- and fat-producers.

As there are fifteen elements entering into the composition of the body, it follows that perfect development can only be had in presence of all these elements in sufficient quantity, and as the framework is largely composed of lime phosphates, it is evident that these must be supplied in the food, and yet as a people we eat largely of that unnatural product, white flour, which contains no lime, while the entire wheat kernel does contain all the fifteen elements of which the body is composed and in about the same proportions, and for this reason it may be called the natural food for man. We may as well build a house out of poor materials, poorly put together, and expect it to endure, as to expect the human structure to be what it was intended if it is built of improper food. If we eat improper food we must suffer; there is no escape. The unfortunate condition of our people to-day is a direct result of our present civilization, and as improper food is the foundation and main cause of human ills, it is plainly seen how poorly we of this land of abundance of good things are supplied with proper food. Bread, the so-called "staff of life," is usually made of white commercial flour, and the other food products made therefrom, such as rolls, buns, waffles, pan-cakes, pies, etc., are the chief cause of the physical weakness and nervous disorders of our people. In the light of the present it is little less than criminal to give a child food made of such flour, especially when at the same meal many other starchy foods are supplied, such as rice, potatoes, corn-starch pudding, etc.

A certain per cent. of what we eat should be heat-making food, another per cent. muscle-making, and another per cent. to make hair, teeth, nerves, etc. If any of these are not provided for, there must be a wasting of tissues, which is but another name for consumption. Under these conditions the system cannot successfully combat disease; on the contrary, it invites it. The panacea of all this trouble is found in nature in the form of the wheat berry, which should be given to us in as nearly a natural condition as possible. The present milling process leaves a product (mainly starch) which is so dead that the American people yearly pay millions of dollars to buy yeast, baking powders, soda, etc., for lightening, and lard, butter, and other greasy substances to shorten and revive this disorganized substance into shape to tickle the palate; and then, to counteract the evil effect of this unnatural food, other millions are spent for medicines. There is no violation of nature's plain requirements so senseless and so damaging as to separate the natural allied properties of the whole wheat. The excessive use of white flour products, together with other starchy foods, is doing and has done more harm to the people than any other agency ever has or ever can do. If children have this kind of food largely, the natural result will be weak nerves, rickety bones, weak muscles, and poor teeth, good candidates for physical failures.

We must have something to change this tendency, and the first essential is to select proper food products. These are such as are organized during the process of their growth. They are natural foods. Nature made them for a food, and organized the material accordingly. Disorganized mixtures are merely attempts at improving on nature's method and they are unnatural foods. Whole wheat is the original basis. There are other naturally organized foods, but whole wheat is the standard. Whole wheat is the natural food product; it grows from the seed, and in the process of growth it extracts in a naturally organized state from the earth and air the properties which when properly cooked make bread both light and short without any foreign aid, as well as to thoroughly nourish every element of the body. There are, of course, other grain foods, many of great dietetic value, but none other so perfect.

The history of all ages and all countries proves that the best-developed men or women were those who lived during the early periods in the development of the countries when naturally organized food was the diet of the people, and that with the progress of time and a supposedly superior dietary came the weaknesses and ills which are so prevalent to-day.

In the mechanical process of making into shreds none of the original elements are lost, and no foreign substance enters into it. It retains all the original constituents of the wheat berry, is simply whole wheat, all wheat, and nothing but wheat. It is a standard food put up in convenient form. Nothing has ever been produced like it. It needs no shortening, yeast, or other chemicals. It can be used with other naturally organized foods for every meal of the day, and while it supplies all the elements of the body under normal conditions, its proportions may be readily changed to suit such altered conditions.

